# Porcine bile acids promote the utilization of fat and vitamin A under low-fat diets

**DOI:** 10.3389/fnut.2022.1005195

**Published:** 2022-09-28

**Authors:** Bowen Yang, Shimeng Huang, Ning Yang, Aizhi Cao, Lihong Zhao, Jianyun Zhang, Guoxian Zhao, Qiugang Ma

**Affiliations:** ^1^College of Animal Science and Technology, Hebei Agricultural University, Baoding, China; ^2^State Key Laboratory of Animal Nutrition, College of Animal Science and Technology, China Agricultural University, Beijing, China; ^3^National Engineering Laboratory for Animal Breeding and Key Laboratory of Animal Genetics, Breeding and Reproduction, College of Animal Science and Technology, China Agricultural University, Beijing, China; ^4^Dezhou Key Laboratory for Applied Bile Acid Research, Shandong Longchang Animal Health Product Co., Ltd., Dezhou, China

**Keywords:** bile acids, laying hens, fat absorption, fat-soluble vitamins, serum immunity, serum antioxidant capacity

## Abstract

Fat-soluble vitamin malabsorption may occur due to low dietary fat content, even in the presence of an adequate supply of fat-soluble vitamins. Bile acids (BAs) have been confirmed as emulsifiers to promote fat absorption in high-fat diets. However, there are no direct evidence of exogenous BAs promoting the utilization of fat-soluble vitamins associated with fat absorption *in vitro* and *in vivo*. Therefore, we chose laying hens as model animals, as their diet usually does not contain much fat, to expand the study of BAs. BAs were investigated *in vitro* for emulsification, simulated intestinal digestion, and release rate of fat-soluble vitamins. Subsequently, a total of 450 healthy 45-week-old Hy-Line Gray laying hens were chosen for an 84-day feeding trial. They were divided into five treatments, feeding diets supplemented with 0, 30, 60, 90, and 120 mg/kg BAs, respectively. No extra fat was added to the basic diet (crude fat was 3.23%). *In vitro*, BAs effectively emulsified the water-oil interface. Moreover, BAs promoted the hydrolysis of fat by lipase to release more fatty acids. Although BAs increased the release rates of vitamins A, D, and E from vegetable oils, BAs improved for the digestion of vitamin A more effectively. Dietary supplementation of 60 mg/kg BAs in laying hens markedly improved the laying performance. The total number of follicles in ovaries increased in 30 and 60 mg/kg BAs groups. Both the crude fat and total energy utilization rates of BAs groups were improved. Lipase and lipoprotein lipase activities were enhanced in the small intestine in 60, 90, and 120 mg/kg BAs groups. Furthermore, we observed an increase in vitamin A content in the liver and serum of laying hens in the 60, 90, and 120 mg/kg BAs groups. The serum IgA content in the 90 and 120 mg/kg BAs groups was significantly improved. A decrease in serum malondialdehyde levels and an increase in glutathione peroxidase activity were also observed in BAs groups. The present study concluded that BAs promoted the absorption of vitamin A by promoting the absorption of fat even under low-fat diets, thereupon improving the reproduction and health of model animals.

## Introduction

Fat-soluble vitamin deficiency is plaguing millions of people in low-income countries, especially among women and children (1). The absorption of fat-soluble vitamins is intimately linked with the fat in the diet (2, 3). However, people in these underdeveloped areas lack the opportunity to consume adequate fat, which leads to the fat-soluble vitamin deficiency. Fat-soluble vitamins, such as vitamins A, D, and E, are essential for human and animal life. For example, vitamin A has a positive effect on growth, development, immunity, and vision (4). Vitamin E plays a critical role in promoting and maintaining reproductive function, promoting follicle development and progesterone secretion, and also has strong antioxidant capacity (5). Vitamin D maintains calcium homeostasis and prevents fractures (6). Therefore, fat-soluble vitamin deficiency causes a decline in fertility and immunity, and causes a series of diseases such as xerophthalmia, anemia, and rickets. Laying hens are a very promising animal model to study the absorption of fat-soluble vitamins. Generally, laying hens’ diets are based on plant ingredients, and do not contain much fat (crude fat content is about 3%). Lower fat content limits the absorption of fat-soluble vitamins, although their supplementation is usually sufficient in the diet. Therefore, improving the absorption of fat will be an effective means of nutritional regulation to improve the absorption of fat-soluble vitamins.

The absorption of fat is inseparable from bile acids (BAs). BAs are the general term for a series of cholanoic acids, mainly found in the bile of animals. BAs are the major products of cholesterol catabolism and play a crucial role in lipid absorption and metabolism (7). Structurally, BAs molecules contain both hydrophilic hydroxyl and carboxyl groups, as well as hydrophobic methyl groups and hydrocarbon cores (8). Furthermore, the steric coordination of hydroxyl and carboxyl groups of BAs are all α-type, so the main configuration of BAs molecules has hydrophilic and hydrophobic sides, resulting in the characteristics of interface-active molecules (9). *In vitro* studies have shown that BAs can not only emulsify fat, but also promote the hydrolysis of fat by lipase (10–13). Previous scholars have proposed that BAs do not act like “double-sided tape” for lipase, but more like an “anchor” (14). Many researchers believed that with the help of BAs or bile salts, fats could be hydrolyzed to more fatty acids by the action of lipase (15). In animal trails, BAs were found to be effectively improve fat absorption by promoting the pancreatic lipase activity in high-fat diets (16).

Although there have been some studies on the promotion of fat absorption by BAs in animals under high-fat diets (16–18), direct evidence for their effect on the absorption of fat-soluble vitamins under low-fat diets is still lacking. Therefore, we chose an alternative to higher-cost pure BAs, which is derived from pig bile. This porcine BAs have the advantages of clear composition, stable properties and lower price. We hypothesized that BAs had both the functions of emulsifying and promoting lipase activity, and through this combined effect, promoted the absorption of fat-soluble vitamins in model animals fed with low-fat diets.

## Materials and methods

### Experimental materials

The porcine BAs used in the *in vitro* and *in vivo* experiments were all from Shandong Longchang Co., Ltd (Dezhou, China). The porcine BAs are composed of the following components: 7.99% of hyocholic acid, 19.65% of chenodeoxycholic acid, and 70.88% of hyodeoxycholic acid. The lipase used in the *in vitro* experiment was from porcine pancreas (OKA Biotechnology Co., Ltd., Beijing, China). The vitamin A used in this study was in the form of retinol (89%; Virtue-Clara, Beijing, China). And the vitamin D used in this study was in the form of ergocalciferol (98%; Macklin Inc., Shanghai, China). The vitamin E used in this study was in the form of α-tocopherol (98%; Solarbio Co., Ltd., Beijing, China).

### *In vitro* experiment

#### Emulsification

A total of 10 g of soybean oil was weighed and placed in beakers, and 0, 400, and 800 mg of BAs were added, respectively. Then, 100 mL of PBS was added to the beakers of each treatment group. After stirring in a magnetic stirrer for 10 min, the liquid was left to stand for 3 min to observe the stratification of the liquid level.

#### *In vitro* lipolysis model

We selected two main kinds of plant oil, soybean oil and corn oil, which often contained in laying hen diets, to measure the lipase-boosting effect of BAs. Since fat is hydrolyzed into fatty acids and glycerol by lipase, we used acid value to measure the fat hydrolysis. A total of 5 g of soybean oil or corn oil was weighed and placed in conical flasks. Then, 300 mg of lipase, 25 mL of PBS, 0, 5, 10, 15, 20, 25, 30, 35, 40, and 45 mg of BAs were added to the conical flasks, respectively. The pH of the solution was adjusted to 6.8 with hydrochloric acid, and then placed in a shaking water bath (180 rpm) to react at 40°C for 3.5 h. After cooling to room temperature, we added 2∼3 drops of phenolphthalein indicator solution in the mixed solution and then titrated with the potassium hydroxide (KOH) standard titration solution (0.05 mol/L), until it appeared reddish and did not fade within 30 s as the end point. To differentiate single BAs and the combined effects of BAs and lipase, we also established lipase-free groups. Three parallel experiments were performed in each group. The calculation formula of acid value is as follows:


$$\text{Acid}\,\text{Value}=\frac{V\times c\times 56.11}{m}$$

Where: *V* is the volume of KOH standard titration solution consumed by the sample, mL; *c* is the actual concentration of KOH standard titration solution, mol/L; *m* is the mass of the sample, g; 56.11 is the mass of KOH equivalent to 1.0 mL of KOH standard titration solution [c(KOH) = 1.00 mol/L], mg.

#### Release rate of fat-soluble vitamins

Firstly, quantitative amounts of vitamins A, D, and E were dissolved in soybean oil or corn oil. Subsequently, lipolysis experiment was carried out according to the aforementioned method. After the lipolysis, the solution was placed in a separatory funnel and left to stand until the layers were separated, and the aqueous layers was separated. The contents of vitamin A, D, and E in the aqueous phase were determined by high performance liquid chromatography with reference to the previous method (19). The vitamin release rate was calculated according to the following formula:


$$\text{Release}\,\text{Rate}=\frac{C_{t}}{C_{\infty }}\times 100$$

Where: *C*_*t*_ is vitamin concentration in aqueous phase; *C*_∞_ is the theoretical maximum concentration of vitamin released in micelles.

### *In vivo* experiment

#### Animals

The protocol of the animal experiment was permitted by the Institutional Animal Care and Use Committee of China Agricultural University (grant No. AW16129102-2; Beijing, China). A total of 450 healthy 45-week-old Hy-Line Gray laying hens were raised in cages. Before the experiment, the hens were reared 7 days in advance to adapt to the experimental environment. Temperature (26°C), humidity (65%), and light (16L: 8D) were accurately controlled throughout the experiment, and hens were allowed to eat and drink *ad libitum*.

#### Design and diets

Hens were randomly divided into five treatment groups with six replicates of 15 birds per replicate. The five treatment groups were fed with: corn soybean meal diets supplemented with 0, 30, 60, 90, and 120 mg/kg porcine BAs, respectively. No extra fat was added to the basic diet (crude fat was 3.23%). The whole experimental period was 84 days, and the pre-feeding period was 7 days. The diet is formulated according to Nutrition Requirement of Chicken in China (NY/T 33-2004) to meet the nutritional requirements. See Table 1 for basic diet formula.

**TABLE 1:** Composition and nutrient level of basal diet (as-fed basis).

Ingredient (%)	Content	Nutrient	Nutrient level
Corn	56.40	AME, Kcal/kg	2650.51
Soybean meal	25.00	Crude protein^3^ (%)	15.20
Dicalcium phosphate	1.50	Digestible Met (%)	0.40
Limestone	8.30	Digestible Lys (%)	0.82
Sodium chloride	0.30	Ca (%)	3.56
DL-methionine	0.17	Total P (%)	0.54
Cornstarch	8.00	Non-phytate P (%)	0.35
Vitamin premix^1^	0.03	Crude fat (%)	3.23
Mineral premix^2^	0.30		
Total	100		

AME, avian metabolic energy. ^1^The vitamin premix supplied (per kilogram of diet): 8,500 IU of vitamin A (retinol acetate), 3,600 IU of vitamin D_3_, 21 IU of vitamin E (DL-α-tocopherol acetate), 4.2 mg of vitamin K_3_, 3.0 mg of vitamin B_1_, 10.2 mg of vitamin B_2_, 0.9 mg of folic acid, 15 mg of calcium pantothenate, 45 mg of niacin, 5.4 mg of vitamin B_6_, 24 μg of vitamin B_12_ and 150 μg of biotin. ^2^The mineral premix provided (per kilogram of diet): 6.8 mg of Cu (CuSO_4_⋅5H_2_O), 66 mg of Fe (FeSO_4_⋅7H_2_O), 83 mg of Zn (ZnSO_4_⋅7H_2_O), 80 mg of Mn (MnSO_4_⋅H_2_O), 0.6 mg of I (KI) and 0.3 mg of Se (Na_2_SeO_3_). ^3^Nutrient levels are analyzed values.

#### Laying performance

The number and weight of eggs produced in each replicate were recorded at a fixed time every day, and the egg production (%) and average egg weight were calculated. Feed consumption was recorded weekly and average daily feed intake (ADFI) was calculated. Feed conversion ratio (FCR) was calculated weekly based on egg mass and feed intake, expressed as feed-to-egg ratio.

#### Sample preparation

Fecal samples were collected every 24 h during the last three consecutive day at the end of the experiment. Samples were dried at 65°C for 48 h, then exposed to air at room temperature for 24 h to prepare air-dried samples. Air-dried samples were ground through a 425 μm sieve for analysis. On the last day of the experiment, six hens from each treatment group were randomly selected for sample collection. After a 12-h fast, sub-wing vein blood was collected into blood collection tubes. After standing at room temperature for stratification, the serum was separated by centrifugation at 1,500 × *g* for 15 min at 4°C in a centrifuge. After blood collection, euthanasia was performed, and the liver, duodenum, jejunum, and ileum mucosa were collected, quick-frozen in liquid nitrogen, and stored at –80°C. Intact ovaries were removed for follicle sorting and counting. On the last day of the experiment, six eggs were collected from each treatment group, and the yolks were separated and stored at –20°C.

#### Apparent nutrient utilization

Acid-insoluble ash (AIA) was used as an endogenous indicator to determine the total tract nutrients apparent utilization according to the method previously described (20). Crude fat (EE) was determined by Soxhlet extraction method according to AOAC (method 920.39). Total energy is determined by oxygen bomb calorimeter (PARR 6400, Parr Instruments Company, Moline, IL, USA) according to ISO 9831:1998. The calculation formula of apparent nutrient utilization is as follows:


$$\text{Apparent}\,\text{nutrient}\,\text{utilization}=100-\left(100\times \frac{N_{d\,i\,g\,e\,s\,t\,a}\times A\,I\,A_{d\,i\,e\,t}}{N_{d\,i\,e\,t}\times A\,I\,A_{d\,i\,g\,e\,s\,t\,a}}\right)$$

Where: *N*_*digesta*_ is the nutrients concentration of the digesta; *AIA*_*diet*_ is the acid-insoluble ash concentration of the diets; *N*_*diet*_ is the nutrients concentration of the diets; *AIA*_*digesta*_ is the acid-insoluble ash concentration of the digesta.

#### Serum indexes

The immunoglobulin A (IgA), immunoglobulin G (IgG), immunoglobulin M (IgM), malondialdehyde (MDA), glutathione peroxidase (GSH-Px), total superoxide dismutase (T-SOD), vitamin A, vitamin D, and vitamin E in the serum were determined by assay kits. All the kits were purchased from Nanjing Jiancheng Bioengineering Institute (Nanjing, China).

#### Fat-soluble vitamins content in liver and yolk

After saponification and extraction, the contents of fat-soluble vitamins A, D, and E in liver and yolk were determined by high performance liquid chromatography (Agilent 1100-VWD, Agilent Technologies Inc., Santa Clara, CA, USA).

#### Classification and counting of follicles in the ovary

Follicles were classified as large yellow follicles (LYF, 8∼10 mm), small yellow follicles (SYF, 5∼7 mm), large white follicles (LWF, 2∼5 mm), and small white follicles (SWF, <2 mm) according their size. All types of follicles were counting after the classification.

#### Intestinal enzyme activity

The activities of lipase (LPS), lipoprotein lipase (LPL) and content of hormone-sensitive lipase (HSL) in duodenum, jejunum and ileum mucosa were measured by assay kits (Nanjing Jiancheng Bioengineering Institute, Nanjing, China).

### Statistical analysis

IBM SPSS Statistics 19.0 program (SPSS Inc., Chicago, IL, USA) was used to process the analysis. GLM procedure was used to analyze variance. The most suitable regression equation was selected based on the curve fit, and the linear or quadratic *P*-values were given. Multiple comparisons among different treatment groups were tested by Tukey’s test. The difference at *P* ≤ 0.05 was considered statistically significant. The replicate served as the experimental unit for all analysis in this experiment.

## Results

### Emulsification effect of porcine bile acids: *In vitro*

**FIGURE 1:**
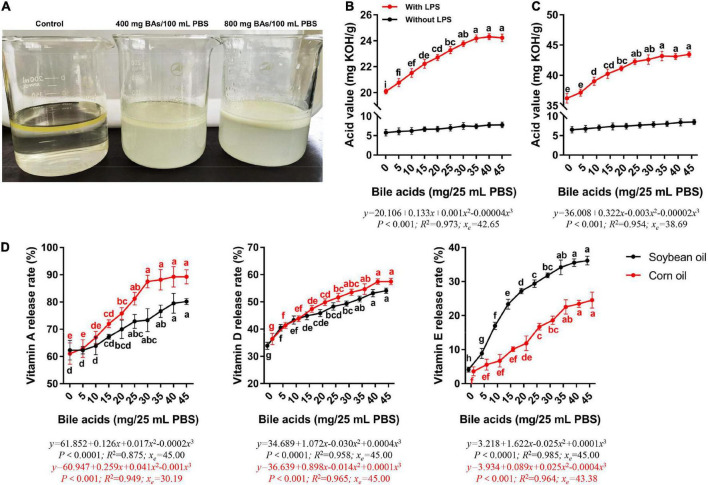
Porcine bile acids promoted emulsification, fat hydrolysis, and release of fat-soluble vitamins *in vitro*. BAs, bile acids. PBS, phosphate buffered solution. KOH, potassium hydroxide. LPS, lipase. **(A)** Emulsification of porcine bile acids. **(B)** Porcine bile acids promoted the hydrolysis of lipase to release more fatty acids in soybean oil. **(C)** Porcine bile acids promoted the hydrolysis of lipase to release more fatty acids in corn oil. **(D)** Porcine bile acids enhanced the release of vitamins A, D and E from fat. **(B–D)** Values are indicated as means ± SD. Different lowercase letters indicate significant differences. The *x*_*e*_ represents the abscissa value at the extremum of the regression equation in this definition domain.

We observed the stratification of the liquid surface after the mixing of water, oil, and BAs (Figure 1A). In the control group, the water and oil layers were obviously separated, and the oil was in the upper layer. The upper layer of oil in the 400 mg BAs group was white foamy, and the yellow color of oil was still seen, indicating that the oil was partially emulsified. When the dosage of BAs reached 800 mg, the upper layer of oil was all in the form of white foam, indicating that the emulsification effect was complete.

### Porcine bile acids promoted lipase hydrolysis of fat: *In vitro*

With the increase of BAs content, the acid value of soybean oil digestive juice increased evidently, and it leveled off until 30 mg of BAs (Figure 1B). Similarly, the acid value of corn oil digestive juice increased with the increase of BAs content (Figure 1C). But the corn oil leveling off occurred in the 25 mg BAs group. Meanwhile, the acid value of soybean oil and corn oil did not change significantly in the lipase-free group. We performed curve fitting and found that the change rule of acid value with the dose of BAs was more in line with the cubic regression both in soybean oil and corn oil (*P* < 0.001). According to the regression equation, it can be known that when the BAs dose is 42.65 and 38.69 mg, the curve of acid value change has an inflection point (extreme value).

### Porcine bile acids aided in fat-soluble vitamin release: *In vitro*

Considering that BAs have both emulsifier properties and the effect of promoting the hydrolysis of fat by lipase, we investigated the release rate of fat-soluble vitamins from oil under the action of BAs. The release rates of the three fat-soluble vitamins increased with the increase of BAs content in both soybean oil and corn oil (Figure 1D). The inflection points for the increased release rates of the three fat-soluble vitamins differed. In both soybean oil and corn oil, the release rate of vitamin A increased with the addition of BAs (*P* < 0.05), while it leveled off in the 25 mg BAs group. Compared with the control group, the release rate of vitamin A in the 25 mg BAs group in soybean oil and corn oil increased by 24.62 and 33.05%, respectively. Similarly, vitamin D and vitamin E also had the same growth trend, but these two vitamins leveled off in the 35 mg BAs group. The vitamin A release rate at the highest value was higher than the other two vitamins in the dosage range of the present study. That is, in soybean oil, the highest release rate of vitamin A (80.20%) was 48.24 and 121.79% higher than that of vitamin D (54.10%) and vitamin E (36.16%), respectively. In corn oil, the maximum release rate of vitamin A (89.26%) was 55.34 and 263.73% higher than that of vitamin D (57.46%) and vitamin E (24.54%), respectively. It seems to suggest that in the presence of BAs, vitamin A is more likely to be released from the oil. Then we carried out curve fitting and found that the cubic regression was more suitable for the change trend of the release rate of these three kinds of fat-soluble vitamins (*P* < 0.001). The release rates of the measured vitamins were monotonically increasing in the range of BAs from 0 to 45 mg.

### Porcine bile acids improved laying performance of laying hens

**FIGURE 2:**
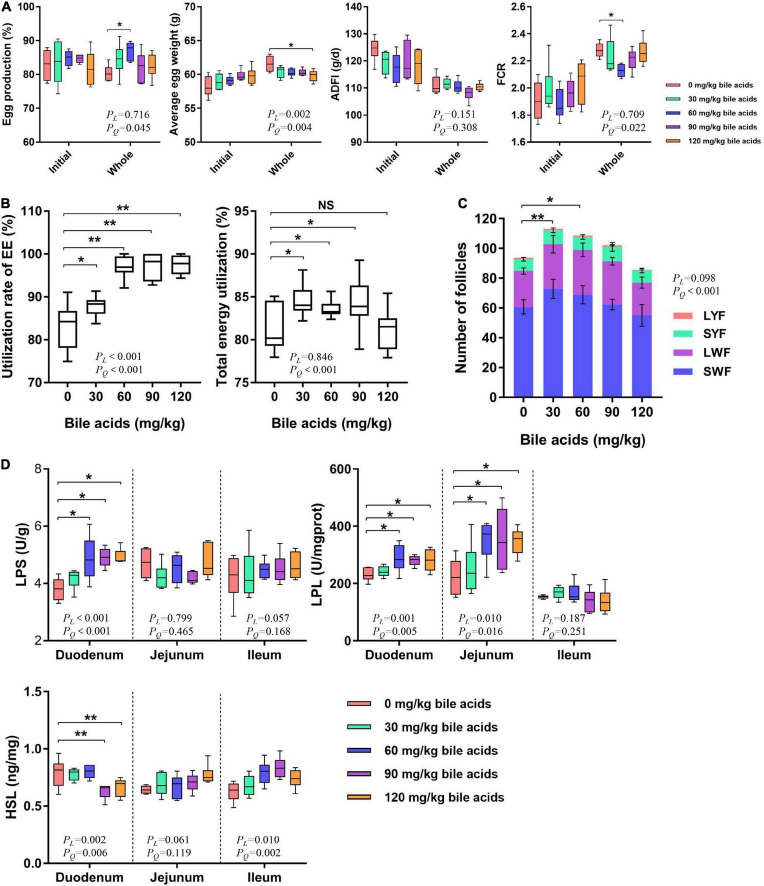
Porcine bile acids improved laying performance by increasing intestinal lipase activity and increasing crude fat utilization. ADFI, average daily feed intake. FCR, feed conversion rate. EE, ether extract. LYF, large yellow follicle. SYF, small white follicle. LWF, large white follicle. SWF, small white follicle. LPS, lipase. LPL, lipoprotein lipase. HSL, hormone-sensitive lipase. *P*_*L*_, linear *P*-value. *P*_*Q*_, quadratic *P*-value. **(A)** Porcine bile acids improved laying performance of laying hens. **(B)** Porcine bile acids enhanced crude fat and total energy utilization. **(C)** Porcine bile acids increased the number of follicles. Values are indicated as means ± SD. **(D)** Porcine bile acids improved the activity of lipase in the small intestine. **(A,B,D)** Each box represents the mean value of six hens each treatment (*n* = 6). Whiskers represent maximum and minimum. Different at *P* < 0.05 is marked as * and *P* < 0.01 is marked as **.

There was no significant difference in the initial production performance of laying hens among the treatment groups (Figure 2A). It can be concluded from Figure 2A that supplementation of 60 mg/kg BAs in the laying hens’ diet improved the laying hen’s egg production rate (*P* < 0.05), while other treatments have no obvious changes. Dietary supplementation of 120 mg/kg of BAs in laying hens reduced average egg weight (*P* < 0.05). However, BAs supplementation had no significant effect on ADFI (*P* > 0.05). This resulted in a significant improvement in the feed conversion ratio of laying hens in the 60 mg/kg BAs group (*P* < 0.05). In view of the significant quadratic regression relationship between laying performance and BAs dose, we calculated the optimal BAs dose according to the regression equation. According to the regression equation of egg production (*y* = 80.742 + 0.150*x*-0.001*x*^2^, *R*^2^ = 0.206) and FCR (*y* = 2.289-0.004*x* + 0.00003*x*^2^, *R*^2^ = 0.246), the optimal amount of BAs supplementation was 65 mg/kg to 75 mg/kg.

### Porcine bile acids increased the utilization of crude fat and energy in laying hens

Crude fat utilization was increased when bile acids were supplemented in the diets of laying hens, with the 60, 90, and 120 mg/kg BAs groups reaching extremely significant levels (*P* < 0.01; Figure 2B). Dietary supplementation of 30, 60, and 90 mg/kg BAs in laying hens significantly improved the energy utilization (*P* < 0.05), but not in the 120 mg/kg BAs group (*P* > 0.05).

### Porcine bile acids increased the number of follicles in laying hens’ ovaries

Dietary supplementation of 30 and 60 mg/kg BAs in laying hens’ diets increased the total number of follicles in ovaries (*P* < 0.05; Figure 2C). However, the number of LYF, SYF, LWF, and SWF did not change significantly.

### Porcine bile acids enhanced relevant lipase activity in the small intestine of laying hens

Dietary supplementation of 60, 90, and 120 mg/kg BAs enhanced LPS activity in duodenal mucosa (*P* < 0.05), but did not affect LPS activity in jejunum and ileum (*P* > 0.05; Figure 2D). In 60, 90, and 120 mg/kg BAs groups, the LPL activity in duodenal and jejunal mucosa was increased (*P* < 0.05), and the change of LPL activity in ileum was not obvious (*P* > 0.05). As for the content of HSL, it was obviously decreased in the duodenal mucosa of 90 and 120 mg/kg BAs groups (*P* < 0.05), but not changed observably in the jejunum and ileum mucosa (*P* > 0.05).

### Porcine bile acids increased fat-soluble vitamin levels in the liver of laying hens

**FIGURE 3:**
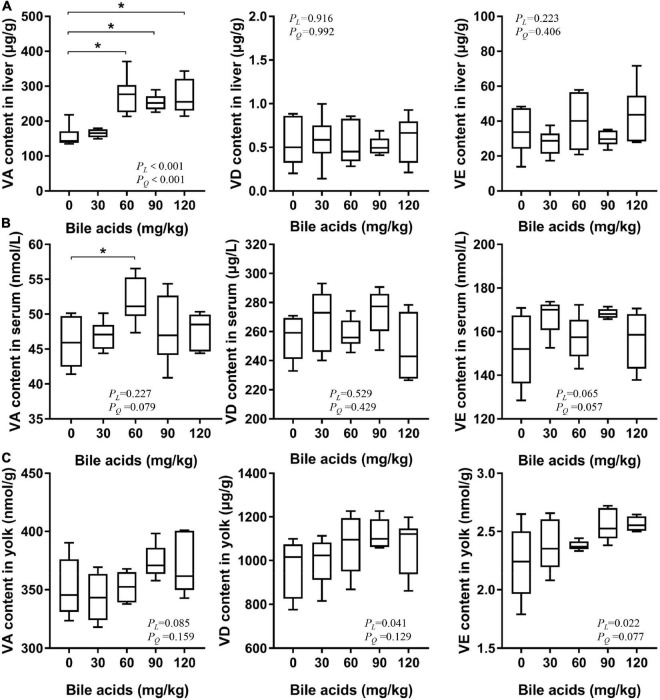
Porcine bile acids enhanced vitamin A absorption in laying hens. VA, vitamin A. VD, vitamin D. VE, vitamin E. *P*_*L*_, linear *P*-value. *P*_*Q*_, quadratic *P*-value. **(A)** Porcine bile acids improved vitamin A content in the liver of laying hens. **(B)** Porcine bile acids improved vitamin A content in serum of laying hens. **(C)** Porcine bile acids did not affect fat-soluble vitamins content in egg yolks. **(A–C)** Each box represents the mean value of six hens each treatment (*n* = 6). Whiskers represent maximum and minimum. Different at *P* < 0.05 is marked as *.

The level of fat-soluble vitamins in the liver reflects the absorption of fat-soluble vitamins by the body. Dietary supplementation of 60, 90, and 120 mg/kg BAs prominently increased the content of vitamin A in the liver of laying hens (*P* < 0.05; Figure 3A). And there were significant linear and quadratic relationships between liver vitamin A content and BAs supplementation (*P* < 0.001). Dietary supplementation of 60, 90, and 120 mg/kg of BAs in laying hens increased the vitamin A content in the liver by 76.61, 62.95, and 73.27%, respectively. This increasing trend is consistent with the results of our *in vitro* experiments, but the growth rate is much higher than that in the *in vitro* experiments. According to the curve estimation, there were significant linear and quadratic relationships between liver vitamin A content and BAs supplementation. Comparing the *R*^2^ values of the two regression equations, it can be judged that this change rule was more suitable for quadratic regression. The regression equation is *y* = 142.027 + 2.139*x*-0.009*x*^2^ (*R*^2^ = 0.544), when *x* = 118.83, *y*_max_ = 269.12. The content of vitamin D and vitamin E was not affected by BAs supplementation (*P* > 0.05). Similarly, we observed that dietary supplementation with 60 mg/kg BAs increased serum vitamin A levels (*P* < 0.05; Figure 3B), while other doses showed no significant change. However, the three vitamins in egg yolks appeared to be increased numerically, but not statistically (Figure 3C).

### Porcine bile acids enhanced serum immune and antioxidant capacity of laying hens

**FIGURE 4:**
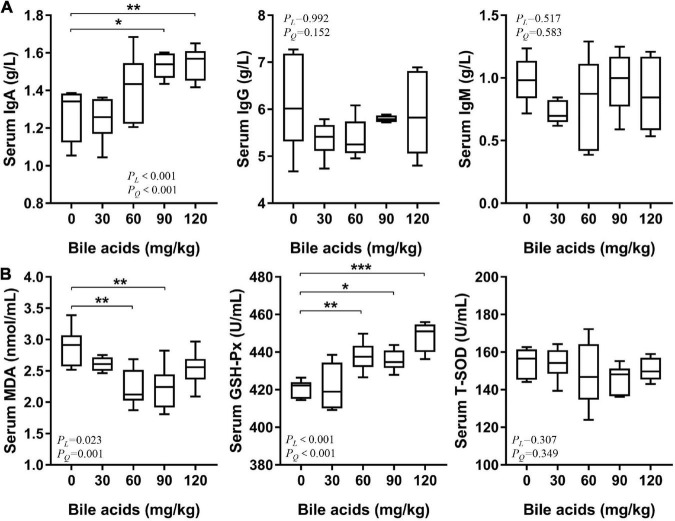
Porcine bile acids enhanced serum immunity and antioxidant capacity of laying hens. IgA/G/M, immunoglobulin A/G/M. MDA, malondialdehyde. GSH-Px, glutathione peroxidase. T-SOD, total superoxide dismutase. *P*_*L*_, linear *P*-value. *P*_*Q*_, quadratic *P*-value. **(A)** Porcine bile acids enhanced serum immunity of laying hens. **(B)** Porcine bile acids enhanced serum antioxidant capacity of laying hens. **(A,B)** Each box represents the mean value of six hens each treatment (*n* = 6). Whiskers represent maximum and minimum. Different at *P* < 0.05 is marked as *, *P* < 0.01 is marked as **, and *P* < 0.001 is marked as ***.

Supplementation of 90 and 120 mg/kg BAs in laying hens’ diets increased serum IgA content (*P* < 0.05), but had no significant effect on IgG and IgM content (Figure 4A). It was observed that serum MDA content decreased in the 60 and 90 mg/kg BAs groups (*P* < 0.05; Figure 4B). The activity of GSH-Px was increased in the 60, 90, and 120 mg/kg BAs groups (*P* < 0.05), but the activity of T-SOD was not affected.

## Discussion

Bile acids are well-known to have surfactant-like properties. The α-type sterically coordinated hydroxyl and carboxyl groups in the molecular structures of BAs allow the specificity of their structures, that is, they have both hydrophobic and hydrophilic ends, which make them have the characteristics of surface active molecules (7). The fat digestion process involves a complex interfacial process, in which BAs act as surfactants to facilitate the adsorption of lipase on the interface. The adsorbed lipase hydrolyzes the fat into fatty acids and glycerol, and then the fatty acids are dissolved by the BAs micelles for absorption by the body. Many *in vitro* experiments have verified the important role of BAs in fat digestion by means of surface tension (21), zeta potential (22) or atomic force microscopy (23). When hydrolyzing fats, lipase needs to be close to the water-oil interface. At this time, the accumulation of too much BAs at the water-oil interface will hinder the contact between lipase and oil, and inhibit the hydrolysis of lipase (24). This may be the reason why the acid value in this study increased with the dose of BAs and then leveled off.

In addition to exerting the effect of surface-active molecules to promote lipase activity, BAs also play an important role in lipase activation. Lipase have special loop structures that cover their hydrolysis sites and prevent the entry of solvents (25). BAs or bile salts can open these loops and bind to lipase to stabilize them at the fat-water interface (26). Lipase must form a “bile acid-colipase-lipase” triplet with BAs and colipase to function (14). It has been reported that low doses (<2 mM) of bile salts have also been shown to open the cap of lipase to activate lipase (27). Also, low doses (0.3 mM) of taurodeoxycholate can greatly improve the stability of lipase (24). Therefore, BAs have a promoting effect on the activity of lipase. These evidences are sufficient to support the results of the present study, that the BAs treatment increased LPS activity in the duodenum of laying hens, which led to an increase in the utilization of crude fat. The improved laying performance of hens benefits from the improved utilization of crude fat. BAs entering the duodenum act first as emulsifiers. Furthermore, BAs promote lipase activity to increase the utilization of crude fat, thereby improving the laying performance of hens. It has been reported that adding fat or increasing metabolizable energy levels to laying hens’ diets can increase egg production (28, 29). But there are few studies on the effects of BAs in laying hens. In our previous studies, we demonstrated that BAs are safe for laying hens and can improve their laying performance, lipid metabolism, and gut microbiota (30, 31). In the study of broilers, it has been proved that the dietary supplementation of 60 mg/kg porcine BAs can improve the growth performance of broilers fed with high-fat diets by increasing the activity of intestinal LPS and LPL (17, 18). These findings can strongly support our results. Most of the previous animal studies on BAs supplementation included additional fat in the diet (16–18). Our study appears to be the first to observe the health-promoting effects of BAs in animal diets without additional fat. This will help to broaden the research perspective on BAs.

As an energy source, fat plays a crucial role in the maturation of oocytes. Studies have shown that surgical removal of the fatty tissue surrounding the ovaries can cause follicular dysplasia and increased atresia in mice (32). And increasing the fat and energy in the feed could help improve follicle development (33). Follicle development is affected by the type and number of fatty acids in the body (34). Fat aids in the restoration of oocyte nuclear maturation, meiosis and acquisition of developmental competence in mouse oocytes via β-oxidation (35–37). In the present study, BAs promoted the absorption of fat in the gut of laying hens and made a great contribution to the development of follicles. And the improvement of follicular development contributed to the improvement of reproductive performance.

Another reason for the improved performance of laying hens may be that BAs promote vitamin A absorption, as evidenced by increased vitamin A in the liver. While promoting fat absorption, BAs can simultaneously promote the absorption of fat-soluble vitamins in feed, which is verified by the present study. The effect of dietary fat on vitamin A absorption has been well documented in human and animal studies (38, 39). Vitamin A plays an important role in growth and development. The *in vivo* experiments in the present study proved that supplementation of BAs increased the vitamin A content in the liver of laying hens by 60–75%. This is approximately equivalent to increasing the amount of vitamin A supplementation in the diet of laying hens. It has been reported that increasing vitamin A levels in diets of laying hens significantly improves feed conversion and egg production (40, 41). These reports are similar to the results of this study. Vitamin A improves the reproductive performance of animals by regulating the expression of ovarian hormone receptors and inhibiting the transcription of apoptotic genes through its active metabolite retinoic acid (42). Also, retinoic acid plays a key role in regulating cell type-specific differentiation during follicular development by mediating the demethylation of the promoter region of luteinizing hormone receptor (43). The dual role of BAs in promoting fat and vitamin A absorption jointly promotes the laying performance of laying hens.

The body relies on chylomicrons secreted by CaCo-2 cells to absorb β-carotene. After adding taurocholic acid, CaCo-2 was promoted to secrete chylomicrons to participate in the absorption of β-carotene (44). In addition, taurocholic acid also aids in the absorption of retinol in the lymph (45). BAs also activate the enzymatic activity of several retinyl ester hydrolases in the small intestine, promoting the release of retinol from retinyl palmitate in the small intestine (46, 47). BAs-induced retinyl ester hydrolase also exists in the liver, which is important for liver uptake and mobilization of vitamin A (48). Similarly, our *in vitro* experiments demonstrated that the presence of BAs increased the release rate of all three fat-soluble vitamins. However, the highest release rate of vitamin A was much higher than that of vitamin D and vitamin E, which leads to the hypothesis that vitamin A could be more readily absorbed in the body than the other two fat-soluble vitamins. These alterations were also consistent with the results of our *in vivo* experiments. *In vivo*, different kinds of fat-soluble vitamins are absorbed at different sites. For example, vitamin A is mainly absorbed in the proximal intestine, while vitamin D is absorbed in the median intestine and vitamin E is absorbed in the distal intestine (49). The preferential absorption of vitamin A competes against the absorption of other fat-soluble vitamins (49). Dietary retinoic acid has also been shown to reduce intestinal absorption of α-tocopherol and promote its oxidation in studies in rats and chicks (50). Moreover, there are also studies showing that the increase of dietary fat supplementation does not promote the absorption of vitamin E (51). This may be the reason why the supplementation of BAs in the diet of laying hens resulted in an increase in the content of vitamin A in the liver, while vitamin D and E did not change significantly in the *in vivo* experiments. In the present study, the growth rate of vitamin A release rate in the *in vitro* experiment compared to the control group was far less than that of vitamin A in the liver of laying hens in the *in vivo* experiment. The *in vitro* experiment supplemented with retinol as the sole source of vitamin A. The *in vivo* diet also contained β-carotene, known as provitamin A, which were converted to vitamin A for storage in the liver (52). While BAs promoted fat absorption, they also promoted the absorption of β-carotene (53), thereby increasing the content of converted vitamin A in the liver. We observed that vitamin A in egg yolks did not change despite BA supplementation increasing both liver and serum vitamin A. For laying hens, both retinol or provitamin A carotenoids are reported to be stored or assembled in the liver, whereas non-provitamin A carotenoids tend to be deposited in eggs (54). A needed in-depth research could revolve around whether BAs can play a role in promoting non-provitamin A carotenoids in egg yolks.

Considering the increased vitamin A content in the liver of laying hens after BAs supplementation, we also detected serum immunoglobulins. The results were promising: IgA levels in the serum of laying hens with elevated hepatic vitamin A were significantly increased. Vitamin A plays an indispensable role in the development of T-helper cells and B-cells (55). In dendritic cells of the gut, retinal is metabolized to retinoic acid by retinal dehydrogenase, which affects B cells and T cells through homing receptors to enhance IgA production (56, 57). It is reported that vitamin A supplementation in the diet of mice can enhance salivary IgA response (58). In studies of respiratory epithelial cells, retinol has been shown to promote IgA secretion by stimulating the expression of interleukin-6 and granulocyte-macrophage colony stimulating factor (59). However, it appears that vitamin A deficiency does not lead to a decrease in plasma IgG and IgM, which has a greater effect on secretory immunoglobulins (4). These studies supported our point that BAs appeared to enhance immunity in laying hens by promoting vitamin A absorption.

In parallel with the serum immunity, we also investigated the effect of dietary BAs supplementation on serum antioxidant capacity of laying hens. It has been reported that after bile duct ligation in mice, the expression of glutathione (GSH) synthase is down-regulated, and the synthesis of glutathione in the body decreases, which leads to liver cell damage, and this symptom can be alleviated by ursodeoxycholic acid treatment (60). This raises the hypothesis that BAs may play some subtle role in promoting GSH synthesis. And GSH-Px is bound to use GSH as a cofactor when GSH-Px exerts its antioxidant effect (61). As in ferroptosis studies, depletion of GSH leads to inactivation of GSH-Px (62). This is consistent with the result of increased GSH-Px activity in the BAs treatment in the present study. Given that BAs also promoted the absorption of vitamin A in laying hens, the increase in GSH-Px activity may also be related to the increase in vitamin A. It has been reported that supplementation of vitamin A in piglets’ diets can increase their serum IgA content and GSH-Px activity (63). MDA is one of the final products of lipid peroxidation, and its content reflects the oxidation of the body (64). With the enhancement of GSH-Px activity *in vivo*, the antioxidant capacity increased, and the content of peroxidation product MDA naturally decreased.

## Conclusion

The present study reinforced the study of BAs, namely that BAs have both emulsifying and lipase-promoting effects. This combined effect improved the body’s absorption of dietary fat and promoted the absorption of vitamin A even under low-fat diets. Due to the improved absorption of vitamin A, the immunity and antioxidant capacity of the model animals were also enhanced. These evidences are sufficient to suggest that BAs supplementation in low-fat diets can be used as an effective means of nutritional regulation to improve the absorption of fat-soluble vitamins and help alleviate vitamin A deficiency in underdeveloped areas.

## Data availability statement

The raw data supporting the conclusions of this article will be made available by the authors, without undue reservation.

## Ethics statement

The animal study was reviewed and approved by Institutional Animal Care and Use Committee of China Agricultural University (grant no. AW16129102-2; Beijing, China).

## Author contributions

QM, AC, SH, NY, and BY designed the study. BY performed the experiments and drafted the manuscript. BY and SH carried out the statistical analysis. QM, SH, NY, AC, LZ, JZ, and GZ helped the revision of this manuscript. NY, QM, and GZ contributed to the funding acquisition, project supervision, and guidance of the present study. All authors read and approved the final manuscript to the published.
